# Expression of Concern: Induction of tyrosine phosphorylated proteins in THP-1 cells by *Salmonella typhimurium, Pasteurella haemolytica* and *Haemophilus influenzae* porins

**DOI:** 10.1093/femspd/ftaf009

**Published:** 2025-08-29

This is an expression of concern regarding: M. Galdiero, M. Vitiello, M. D'Isanto, L. Peluso, M. Galdiero, Induction of tyrosine phosphorylated proteins in THP-1 cells by *Salmonella typhimurium, Pasteurella haemolytica* and *Haemophilus influenzae* porins, *FEMS Immunology & Medical Microbiology*, Volume 31, Issue 2, August 2001, Pages 121–130, https://doi.org/10.1111/j.1574-695X.2001.tb00508.x

Concerns regarding similarities within Figures 2,3,4,5,6,7,8 and 9 were raised on Pub Peer [https://pubpeer.com/publications/B46436ACDFFA4F9617848892ADFFEA] and subsequently were flagged to the Editor-in-Chief in 2025.

The journal attempted to contact the corresponding author but did not receive a response. The journal is publishing this Expression of Concern to notify readers of the concerns whilst the outcome of the investigation is pending and advises readers to examine the details of this study with particular care.

